# Perinatal Mental Health Conditions Among U.S. Active Component Service Women, 2016–2022

**Published:** 2026-02-04

**Authors:** Laura L. Manzo, Clinton Hall, Ilan Harpaz-Rotem, Angela K. Phillips, Julie A. Womack, Joan Combellick

**Affiliations:** Womack Army Medical Center, Ft Bragg, NC: MAJ Manzo; Yale School of Nursing, West Haven, CT: MAJ Manzo, Dr. Combellick, Dr. Womack; Leidos, Inc., San Diego, CA: Dr. Hall; Veterans Administration Connecticut Healthcare System, West Haven, CT: Dr. Combellick, Dr. Harpaz-Rotem, Dr. Womack; Yale University, New Haven, CT: Dr. Harpaz-Rotem; Air Force Medical Command, Falls Church, VA: Lt Col Phillips

## Abstract

Although mental health conditions are the leading underlying cause of maternal mortality, there is limited research on the prevalence of perinatal mental health conditions among active duty service women (ADSW). In this study of live-born deliveries among U.S. ADSW (n=62,729) with pregnancy start and end dates (i.e., dates of last menstrual period and infant delivery, respectively) from October 1, 2016 through December 31, 2021, International Classification of Diseases, 10th Revision, Clinical Modification diagnosis codes were used to identify mental health conditions: trauma and stressor-related disorders, anxiety and panic disorders, depressive disorders, suicidal ideation or attempt, and eating disorders. Data were collected through 1 year postpartum, until December 31, 2022. The prevalence of diagnosed mental health conditions from 1 year prior to pregnancy through 1 year postpartum was 33.8%. Trauma and stressor-related disorders were most prevalent (23.1%), followed by anxiety and panic disorders (16.9%), depressive disorders (14.6%), suicidal ideation or attempt (1.6%), and eating disorders (0.4%). The prevalence of mental health conditions was higher in the postpartum period (22.0%) compared to pregnancy (18.4%) and prior to pregnancy (15.0%). Overall, higher prevalence of these conditions was found among non-Hispanic Black ADSW (37.4%), and those who were unmarried (38.4%), never deployed (34.9%), or in the Army (37.4%) and Navy (36.4%).

**What are the new findings?**

One in 3 active duty service women were diagnosed with a mental health condition in the year preceding pregnancy through 1 year postpartum. Overall, non-Hispanic Black and junior enlisted active duty service women demonstrated higher prevalences of mental health conditions compared to all other racial and ethnic groups and military ranks.

**What is the impact on readiness and force health protection?**

Mental health issues can lead to early returns from deployment, which can adversely affect unit missions and cohesion. Service member retention is linked to mental health, with those who experience mental health conditions less likely to remain in military service. As the proportion of women serving in the military continues to increase, targeted perinatal mental health support and interventions should be prioritized to improve active duty service women's psychological well-being and maintain force readiness.

Most (93.5%) U.S. active duty service women (ADSW) are of childbearing age (18-44 years), averaging 15,000 live births per year. ^1^ From 2017 through 2019, 22.7% of maternal mortality in the general U.S. population was attributable to mental health conditions, including deaths due to suicide or overdose. ^2^ While comparable maternal mortality data are not published for ADSW, during the same period 37.8% of ADSW received a mental health diagnosis during pregnancy or through 1 year postpartum. ^3^

Recent research suggests that deaths from suicide or accidental overdose account for a much larger percentage of pregnancy-associated deaths among ADSW (39.4%) compared to civilian women (8.8–10.9%). ^4^ The increased burden of mental health conditions among ADSW continues after military service, with as many as 46.7% of female veterans reporting perinatal depression, compared to 10% of civilian women. ^5^ Existing research only provides the prevalence of any mental health condition versus specific diagnoses, without estimates for sub-populations of ADSW. This report describes the 2016–2022 prevalence of perinatal mental health conditions among ADSW, with data presented for 5 diagnostic categories: trauma and stressor-related disorders, anxiety and panic disorders, depressive disorders, suicidal ideation or attempt, and eating disorders.

## Methods

### Data sources

This study utilized data from the Department of Defense (DOD) Birth and Infant Health Research (BIHR) program, a population-level surveillance and research database that identifies live births among Military Health System (MHS) beneficiaries. Detailed information on BIHR data and methodologies have been described elsewhere. ^6 , 7^ BIHR includes military personnel data from the Defense Manpower Data Center (DMDC) and administrative medical encounter data from the MHS Data Repository; BIHR data are used to identify and link live births to birth mothers and military sponsors through the Defense Enrollment Eligibility Reporting System, to describe associated demographic and medical characteristics. These data are linked using the unique 10-digit identifiers (i.e., Electronic Data Interchange Personal Identifier) assigned to each person with a direct DOD relationship. Institutional Review Board approval (NHRC.1999.0003) for this study was obtained from the Naval Health Research Center, with informed consent waived in accordance with criteria set forth by 32 Code of Federal Regulations Section 219.116(d).

### Study population

The source population for this study included all live-born deliveries among ADSW captured in BIHR data with pregnancy start and end dates (i.e., dates of last menstrual period, or LMP, and delivery, respectively) from October 1, 2016 through December 31, 2021. This timeframe was selected to include medical data that were captured exclusively after the transition to the International Classification of Diseases, 10th Revision, Clinical Modification (ICD-10-CM), which occurred on October 1, 2015. Deliveries were excluded if an ADSW did not have a record of TRICARE enrollment or any medical encounter data for at least 10 of 12 months during both the year preceding pregnancy and year following delivery.

### Mental health conditions

Mental health conditions of interest were identified using ICD-10-CM codes and then grouped into 5 categories: trauma and stressor-related disorders (F43.x), anxiety and panic disorders (F40.x, F41.x), depressive disorders (F32.x, F33.x, F34.x), suicidal ideation or attempt (R45.851, T14.91), and eating disorders (F50.x), in accordance with the *Diagnostic and Statistical Manual of Mental Disorders, Fifth Edition, Text Revision* . ^8^ Conditions were measured within 3 timeframes: pre-pregnancy (year prior to LMP), pregnancy (from LMP to date of delivery), and postpartum (year following date of delivery). For each diagnostic category and time-frame, cases were identified by the presence of diagnosis codes on 1 inpatient or 2 outpatient records on separate dates. This method was selected to improve reliability for capturing true mental health diagnoses and not exclusion or ‘rule out’ diagnoses, which are common with new mental health conditions, as many conditions exhibit overlapping symptoms. Diagnostic categories and timeframes were not mutually exclusive, meaning that ADSW with live-born deliveries could be identified with multiple mental health conditions within multiple mental health diagnostic categories at multiple timeframes in the study period. For example, if an ADSW had both an anxiety and depressive disorder diagnosis she would be represented individually in both diagnostic categories (anxiety or panic disorders and depressive disorders).

To provide the prevalence of diagnosed mental health conditions pre-pregnancy through 1 year postpartum, an overall composite variable was created to identify deliveries that met criteria for any mental health condition of interest during any timeframe. Variables were created to identify deliveries with any diagnosed mental health conditions of interest within each timeframe (pre-pregnancy, pregnancy, postpartum). To evaluate the prevalence of specific mental health conditions, variables were created for each diagnostic category (trauma/stress, anxiety/panic, depressive, suicidal ideation/attempt, eating disorders) assessed over the entire study period and within each timeframe (pre-pregnancy, pregnancy, postpartum). We also created a co-morbid mental health condition variable that summed the number of diagnosed mental health conditions (from the 5 diagnostic categories of interest) for each individual throughout the study period (1 year pre-pregnancy through 1 year postpartum). The count ranged 0–5 and was categorized as 0, 1, 2, or 3+.

### Covariates

Demographic and military factors were obtained from DMDC files corresponding to the month of delivery. Variables included racial or ethnic group (i.e., American Indian or Alaska Native, Asian, Hispanic, multiracial, Native Hawaiian/Pacific Islander, non-Hispanic Black, non-Hispanic White, unknown), age at infant delivery (<20, 20-24, 25-29, 30-34, 35+ years), marital status (married, unmarried/unknown), military rank and pay grade (junior enlisted [E1-E4], mid-/senior enlisted [E5-E9], officer/warrant officer [O1-O10/W01-W05]), branch of service (Army, Navy, Air Force, Marine Corps, Coast Guard), and deployment history prior to infant delivery (ever deployed, never deployed). Deployment history was limited to deployments in support of post-September 11, 2001 (9/11) operations, predominately in or near the Middle East.

### Statistical analysis

Frequencies and percentages were used to describe the prevalence of mental health conditions during the study period, and by demographic and military characteristics. Confidence intervals (CIs) were also calculated to assess differences between subgroups. Prevalence was not calculated for subgroups with less than 30 cases. Prevalence was calculated for the mental health conditions overall and by specific diagnostic category; measures were calculated throughout the entire study (1 year pre-pregnancy through 1 year postpartum) and by specific timeframe. All data management and statistical analyses were performed using SAS, Version 9.4 (SAS Institute Inc., Cary, NC).

## Results

### Analytic population

The source population included 62,729 live-born deliveries among 54,471 unique ADSW. After excluding deliveries among ADSW with less than 10 of 12 months of either TRICARE enrollment or medical encounter data before and after pregnancy, the final analytic cohort comprised 56,371 deliveries among 49,262 unique ADSW (89.9% of source population). Excluded deliveries were more likely to be among ADSW younger than age 20 years, of junior enlisted rank, and in the Marine Corps.

### Prevalence of any mental health condition

Overall, 33.8% of deliveries were among ADSW diagnosed with at least 1 mental health condition of interest at any time during the study period **(Table 1)** . Deliveries to non-Hispanic Black ADSW had the highest prevalence of any mental health condition (37.4%; 95% CI 36.6, 38.2) compared to all other racial and ethnic groups (range 23.6–34.3%). A higher prevalence of mental health conditions was found among deliveries to unmarried versus married ADSW (38.4%; 95% CI 37.5, 39.2 vs. 32.5%; 95% CI 32.1, 33.0). The prevalence of mental health conditions was lower among deliveries to officers (20.8%; 95% CI 20.1, 21.6) compared to those among junior enlisted (38.5%; 95% CI 37.3, 39.6) and mid- or senior enlisted ADSW (36.6%; 95% CI 36.1, 37.0).

**TABLE 1.** Prevalence of Perinatal Mental Health Conditions Among Live Birth Deliveries, Active Component, U.S. Armed Forces, Department of Defense Birth and Infant Health Research Program Data, 2016–2022

	Live Births (n)	Any Mental Health Condition ^a^			
		No.	%	95% CI Lower Limit	95% CI Upper Limit
Total	56,371	19,077	33.8	33.5	34.2
Race and ethnicity					
American Indian, Alaska Native	591	192	32.5	28.7	36.3
Asian, non-Hispanic	2,262	606	26.8	25.0	28.6
Black, non-Hispanic	13,709	5,130	37.4	36.6	38.2
Hispanic	10,946	3,535	32.3	31.4	33.2
Multi-racial, non-Hispanic	2,562	878	34.3	32.4	36.1
Native Hawaiian, Pacific Islander	828	195	23.6	20.7	26.4
White, non-Hispanic	24,146	8,086	33.5	32.9	34.1
Unknown	1,327	455	34.3	31.7	36.8
Age, *y*					
<20	468	165	35.3	30.9	40.0
20–24	19,345	7,261	37.5	36.9	38.2
25–29	17,281	5,929	34.3	33.6	35.0
30–34	12,267	3,518	28.7	27.9	29.5
35+	7,010	2,204	31.4	30.4	32.5
Marital status					
Married	43,595	14,176	32.5	32.1	33.0
Unmarried, unknown	12,776	4,901	38.4	37.5	39.2
Military rank					
Junior enlisted	6,872	2,645	38.5	37.3	39.6
Mid-, senior enlisted	38,926	14,230	36.6	36.1	37.0
Officer	10,573	2,202	20.8	20.1	21.6
Service branch					
Army	18,668	6,979	37.4	36.7	38.1
Navy	15,969	5,817	36.4	35.7	37.2
Air Force	16,207	4,685	28.9	28.2	29.6
Marine Corps	4,131	1,279	31.0	30.0	32.4
Coast Guard	1,396	317	22.7	20.5	24.9
Deployment history ^b^					
Ever deployed	19,027	6,064	31.9	31.2	32.5
Never deployed	37,344	13,013	34.9	34.4	35.3
Stage of pregnancy					
Pre-pregnancy	56,371	8,444	15.0	14.7	15.3
During pregnancy	56,371	10,376	18.4	18.1	18.7
Postpartum	56,371	12,405	22.0	21.7	22.4
Diagnoses of mental health conditions					
1	56,371	10,085	17.9	17.6	18.2
2	56,371	5,614	10.0	9.7	10.2
3+	56,371	3,378	6.0	5.8	6.2

Abbreviations: No., number; CI, confidence interval; *y* , years.

a Composite variable which includes all 5 mental health diagnostic categories (trauma / stressor-related disorders, anxiety / panic disorders, depressive disorders, suicidal ideation / attempt, eating disorders).

b Limited to deployments in support of post-9/11 operations, predominately in and around Middle East region.

ADSW in the Army had the highest prevalence of mental health conditions (37.4%; 95% CI 36.7, 38.1), followed by those in the Navy (36.4%; 95% CI 35.7, 37.2), compared to deliveries among ADSW in the Air Force, Coast Guard, and Marine Corps (range 22.7–31.0%). Those who had never deployed had a higher prevalence of mental health conditions compared to those who had a history of deployment (34.9%; 95% CI 34.4, 35.3 vs. 31.9%; 95% CI 31.2, 32.5), where the definition of deployment was limited to support of post-9/11 operations.

The prevalence of any diagnosed mental health condition increased over time during the perinatal period, from 15.0% (95% CI 14.7, 15.3) in the year prior to pregnancy to 18.4% (95% CI 18.1, 18.7) during pregnancy to 22.0% (95% CI 21.7, 22.4) in the year following pregnancy. Throughout the study cohort, 17.9% (95% CI 17.6, 18.2) of deliveries were to ADSW with 1 diagnosed mental health condition, 10.0% (95% CI 9.7, 10.2) were to ADSW with 2 mental health conditions, and 6.0% (95% CI 5.8, 6.2) were to ADSW with 3 or more mental health conditions. Of those diagnosed with any of the 5 mental health conditions during the study period, 29.4% had at least 2 diagnoses, and 17.7% had 3 or more diagnoses (data not shown).

### Prevalence of specific mental health conditions

Throughout the entire study period, the most commonly diagnosed mental health conditions were trauma and stressor-related disorders (23.1%; 95% CI 22.8, 23.5), followed by anxiety and panic disorders (16.9%; 95% CI 16.6, 17.3), depressive disorders (14.6%; 95% CI 14.4, 14.9), suicidal ideation or attempt (1.6%; 95% CI 1.5, 1.7), and eating disorders (0.4%; 95% CI 0.3, 0.4) **(Table 2)** . Similar to the overall prevalence of mental health conditions, when examined by specific diagnostic category, a higher prevalence of all diagnoses was found in deliveries of ADSW who were unmarried, of enlisted rank, in the Army or Navy, or who had never deployed.

**TABLE 2.** Prevalence of Specific Perinatal Mental Health Conditions, Among Live Birth Deliveries, Active Component, U.S. Armed Forces, Department of Defense Birth and Infant Health Research Program Data, 2016–2022

	Total (n)	Trauma, Stressor				Anxiety, Panic				Depression				Suicidal Ideation, Attempt				Eating Disorder			
		No.	%	95% CI Lower Limit	95% CI Upper Limit	No.	%	95% CI Lower Limit	95% CI Upper Limit	No.	%	95% CI Lower Limit	95% CI Upper Limit	No.	%	95% CI Lower Limit	95% CI Upper Limit	No.	%	95% CI Lower Limit	95% CI Upper Limit
Total	56,371	13,028	23.1	22.8	23.5	9,548	16.9	16.6	17.3	8,251	14.6	14.4	14.9	879	1.6	1.5	1.7	216	0.4	0.3	0.4
Race and ethnicity																					
American Indian, Alaska Native	591	128	21.7	18.3	25.0	100	16.9	13.9	19.9	87	14.7	11.9	17.6	^a^	^a^	^a^	^a^	^a^	^a^	^a^	^a^
Asian, non-Hispanic	2,262	428	18.9	17.3	20.5	252	11.1	9.8	12.4	243	10.5	9.5	12.0	^a^	^a^	^a^	^a^	^a^	^a^		a
Black, non-Hispanic	13,709	3,765	27.5	26.7	28.2	2,167	15.8	15.2	16.4	2,338	17.1	16.4	17.7	303	2.2	2.0	2.5	43	0.3	0.2	0.4
Hispanic	10,946	2,475	22.6	21.8	23.4	1,730	15.8	15.1	16.5	1,578	14.4	13.8	15.1	186	1.7	1.5	1.9	40	0.4	0.3	0.5
Multi-racial, non-Hispanic	2,562	585	22.8	21.2	24.5	467	18.2	16.7	19.7	407	15.9	14.5	17.3	34	1.3	0.9	1.8	^a^	^a^	^a^	^a^
Native Hawaiian, Pacific Islander	828	142	17.2	14.6	19.7	67	8.1	6.2	10.0	82	9.9	7.9	11.9	^a^	^a^	^a^	^a^	^a^	^a^	^a^	^a^
White, non-Hispanic	24,146	5,216	21.6	21.1	22.1	4,524	18.7	18.2	19.2	3,331	13.8	13.4	14.2	285	1.2	1.0	1.3	108	0.5	0.4	0.5
Unknown	1,327	289	21.8	19.6	24.0	241	18.2	16.1	20.2	185	13.9	12.1	15.8	^a^	^a^	^a^	^a^	^a^	^a^	^a^	^a^
Age, *y*																					
<20	468	116	24.8	20.9	28.7	68	14.5	11.3	17.7	65	13.9	10.8	17.0	^a^	^a^	^a^	^a^	^a^	^a^	^a^	^a^
20–24	19,345	5,174	26.8	26.1	27.4	3,420	17.7	17.1	18.2	3,243	16.8	16.2	17.3	575	3.0	2.7	3.2	89	0.5	0.4	0.6
25–29	17,281	4,010	23.2	22.6	23.8	3,052	17.7	17.1	18.2	2,551	14.8	14.2	15.3	191	1.1	1.0	1.3	58	0.3	0.3	0.4
30–34	12,267	2,264	18.5	17.8	19.1	1,854	15.1	14.5	15.8	1,448	11.8	11.2	12.4	65	0.5	0.4	0.7	38	0.3	0.2	0.4
35+	7,010	1,464	20.9	19.9	21.8	1,154	16.5	15.6	17.3	944	13.5	12.7	14.3	38	0.5	0.4	0.7	^a^	^a^	^a^	^a^
Marital status																					
Married	43,595	9,516	21.8	21.4	22.2	7,219	16.6	16.2	16.9	5,993	13.8	13.4	14.1	574	1.3	1.2	1.4	151	0.4	0.3	0.4
Unmarried, unknown	12,776	3,512	27.5	26.7	28.3	2,329	18.2	17.6	18.9	2,258	17.7	17.0	18.3	305	2.4	2.1	2.7	65	0.5	0.4	0.6
Rank																					
Junior enlisted	6,872	1,897	27.6	26.6	28.7	1,215	17.7	16.8	18.6	1,195	17.4	16.5	18.3	234	3.4	3.0	3.8	33	0.5	0.3	0.6
Mid-, senior enlisted	38,926	9,785	25.1	24.7	25.6	7,227	18.6	18.2	19.0	6,289	16.2	15.8	16.5	607	1.6	1.4	1.7	148	0.4	0.3	0.4
Officer	10,573	1,346	12.7	12.1	13.4	1,106	10.5	9.9	11.0	767	7.3	6.8	7.8	38	0.4	0.3	0.5	35	0.3	0.2	0.4
Service branch																					
Army	18,668	5,088	27.3	26.6	27.9	3,170	17.0	16.4	17.5	2,947	15.8	15.3	16.3	429	2.3	2.1	2.5	70	0.4	0.3	0.5
Navy	15,969	3,918	24.5	23.9	25.2	3,073	19.2	18.6	19.9	2,751	17.1	16.6	17.8	215	1.4	1.2	1.5	69	0.4	0.3	0.5
Air Force	16,207	2,928	18.1	17.5	18.7	2,515	15.5	15.0	16.1	1,865	11.5	11.0	12.0	169	1.0	0.9	1.2	44	0.3	0.2	0.4
Marine Corps	4,131	920	22.3	21.0	23.5	607	14.7	13.6	15.8	554	13.4	12.4	14.5	63	1.5	1.1	1.9	28	0.7	0.4	0.9
Coast Guard	1,396	174	12.5	10.7	14.2	183	13.1	11.3	14.9	134	9.6	8.1	11.1	^a^	^a^	^a^	^a^	^a^	^a^	^a^	^a^
Deployment history ^b^																					
Ever deployed	19,027	4,077	21.4	20.8	22.0	3,140	16.5	16.0	17.0	2,654	14.0	13.5	14.4	196	1.0	0.9	1.2	78	0.4	0.3	0.5
Never deployed	37,344	8,951	24.0	23.5	24.4	6,408	17.2	16.8	17.5	5,597	15.0	14.6	15.4	683	1.8	1.7	2.0	138	0.4	0.3	0.4
Stage of pregnancy																					
Pre-pregnancy	56,371	5,819	10.3	10.1	10.6	3,074	5.5	5.3	5.6	2,608	4.6	4.5	4.8	343	0.6	0.5	0.7	99	0.2	0.1	0.1
During pregnancy	56,371	5,279	9.4	9.1	9.6	5,501	9.8	9.5	10.0	4,703	8.3	8.1	8.6	219	0.4	0.3	0.4	97	0.2	0.1	0.2
Postpartum	56,371	7,999	14.2	13.9	14.5	4,961	8.8	8.6	9.0	4,422	7.8	7.6	8.1	380	0.7	0.6	0.7	105	0.2	0.2	0.2

Abbreviations: n, number; No., number; CI, confidence interval; *y* , years.

a Frequencies and 95% CIs were not calculated for cells with less than 30.

b Limited to deployments in support of post-9/11 operations, predominately in and around Middle East region.

For trauma and stressor-related disorders, deliveries to non-Hispanic Black ADSW had the highest prevalence (27.5%; 95% CI 26.7, 28.2) compared to all other racial and ethnic groups (range 17.2–22.8%). By age, the lowest prevalence for trauma and stress-related disorders was in deliveries among ADSW ages 30-34 years (18.5%; 95% CI 17.9, 19.1) compared to all other age groups (range 20.9–26.8%). The highest prevalence of trauma and stressor-related disorders was seen postpartum (14.2%; 95% CI 13.9, 14.5), compared to pre-pregnancy (10.3%; 95% CI 10.1, 10.6) and during pregnancy (9.4%; 95% CI 9.1, 9.6).

For anxiety and panic disorders, the lowest prevalence was found among deliveries to Native Hawaiian or Pacific Islander (8.1%; 95% CI 6.2, 10.0) and non-Hispanic Asian (11.1%; 95% CI 9.8, 12.4) ADSW compared to all other racial or ethnic groups (range 15.8–18.7%). Deliveries to ADSW in the Navy had the highest prevalence (19.2%; 95% CI 16.8, 19.9) compared to all other service branches (range 13.1–17.0%).

For depressive disorders, higher prevalence was found among deliveries to junior enlisted (17.4%; 95% CI 16.5, 18.3) and mid- or senior enlisted ADSW (16.2%; 95% CI 15.8, 16.5) compared to officers (7.3%; 95% CI 6.8, 7.8). The lowest prevalence of depressive disorders was seen pre-pregnancy (4.6%; 95% CI 4.5, 4.8) compared to during pregnancy (8.3%; 95% CI 8.1, 8.6) and postpartum (7.8%; 95% CI 7.6, 8.1).

For suicidal ideation or attempt, junior enlisted ADSW prevalence (3.4%; 95% CI 3.0, 3.8) was 8.5 times the prevalence among officers (0.4%; 95% CI 0.3, 0.5) and 2 times prevalence in mid- and senior enlisted ADSW (1.6%; 95% CI 1.4, 1.7).

For eating disorders, there were no significant differences in prevalence by demographic or military characteristics as well as timeframe.

## Discussion

In this study of live-born deliveries among ADSW, 1 in 3 were diagnosed with a mental health condition 1 year prior to pregnancy through 1 year postpartum. Of those diagnosed with a mental health condition, 1 in 4 were diagnosed with a trauma and stressor-related disorder, which existing research has linked to an increase in suicide risk, particularly among women. ^9 - 12^ Mental health conditions are also associated with adverse pregnancy outcomes, such as pre-term birth and hypertensive disorders of pregnancy, with highest risk in those with trauma or stressor-related disorders. ^13 - 16^ This high prevalence (33.8%) of mental health conditions reveals the potential risk of adverse pregnancy outcomes among ADSW. ^14 , 16 , 17^ To our knowledge, this is the largest study to examine the prevalence of specific mental health conditions before and during the perinatal period among a sample of live births to ADSW. ^3^

There is limited research focused on perinatal mental health conditions among ADSW. Abramovitz and colleagues investigated the prevalence of post-traumatic stress disorder (PTSD) (identified by diagnosis codes) among 134,244 pregnant ADSW from 2007 through 2014, utilizing the same data source (BIHR) as the current study. Abramovitz et al. found that 1.7% of ADSW had a diagnosis of PTSD from the year prior to pregnancy through the end of pregnancy. ^18^ In contrast, this study estimated the prevalence of all trauma or stressor-related disorders, not just PTSD, and found a higher prevalence during prepregnancy (10.3%) and pregnancy (9.4%). A recent study of a nationally representative sample in the U.S. found a prevalence of a trauma or stressor-related disorder during pregnancy of only 0.2%, much lower than reported in this study and existing studies of military populations. ^14^

Andriotti and colleagues utilized TRI-CARE claims data to identify new mental health cases in the 2 years prior to pregnancy, during pregnancy, and 2 years postpartum. ^17^ Andriotti et al. provided limited details, however, for which mental health conditions were included in their study or which specific diagnosis codes were used to identify mental health cases. As in our study, Andriotti et al. found an increase in the prevalence of mental health conditions in the postpartum period (20%) compared to pregnancy (15%). ^17^ Globally, the prevalence of perinatal mental health conditions is estimated to be 10% during pregnancy and 13% postpartum, which is lower than that found by Andriotti and colleagues ^17^ as well as the current study of ADSW. ^19^

A U.S. Government Accountability Office (GAO) report of perinatal mental health conditions among TRICARE beneficiaries, 2017–2019, found that 37.8% of ADSW had a diagnosed mental health condition during pregnancy or in the year postpartum. ^3^ The GAO estimate is higher than this study's estimated 33.8% prevalence, with several methodological differences between the 2 studies. First, the GAO report defined mental health cases by the presence of any mental health ICD-10 code (F01-F99) and only required 1 code, either inpatient or outpatient. The current study limited analysis to 5 categories of mental health conditions defined by the presence of either 1 inpatient ICD-10 code or 2 outpatient ICD-10 codes on separate days. We chose this method to improve reliability of capturing a true diagnosed case versus exclusion or ‘rule out’ diagnoses. Second, the GAO report only included mental health conditions diagnosed during pregnancy through 1 year postpartum, while this study also included mental health diagnoses in the year prior to pregnancy. Finally, this study only included live-born deliveries, while the GAO report included pregnancy losses and stillbirths. Despite these methodological differences, the results from the GAO report and this study are similar: Both found a higher prevalence of mental health conditions in non-Hispanic Black ADSW compared to other racial and ethnic groups, and a higher prevalence among ADSW in the Army and Navy compared to other service branches.

One key difference between the GAO report and this study is that the GAO found a higher prevalence of mental health conditions among ADSW who deployed, while this study found that ADSW who had not deployed had a higher prevalence. To capture deployment history, the GAO report relied on ICD-10 code (Z91.82), while this study obtained deployment data from DMDC limited to deployments in support of post-9/11 operations. This difference in methodology may explain the differing results.

This study also found lower prevalence among Native Hawaiian and Pacific Islander ADSW compared to all other racial and ethnic groups, which conflicts with existing, albeit limited, research. ^20 , 21^ This study's population included 1,419 ADSW who identified as American Indian, Alaska Native, Native Hawaiian, or Pacific Islander, which provided a rare opportunity to evaluate perinatal mental health in these historically under-researched populations. Women of those racial and ethnic groups may face unique barriers to care due to living in remote geographic locations, lack of culturally appropriate screening tools, insufficient cultural congruency with health care providers, and other structural factors that increase their risk of poor health outcomes. ^20^ Those risks may be mitigated by military service, which provides access to health care and stable salaries, both factors that may help explain why rates of perinatal mental health conditions in this study were lower among Native Hawaiian and Pacific Islander ADSW compared to other racial and ethnic groups, including American Indian and Alaska Native service women.

Strengths of this study include the use of a large, population-based dataset of ADSW. All women in this study were employed, with access to health care, which provided a unique opportunity to examine differences in perinatal mental health by socio-demographic characteristics. Another strength of this study is that mental health conditions were evaluated at 3 distinct points in time—from 1 year prior to pregnancy, during pregnancy, and at 1 year postpartum—which allowed assessment of prevalence over time. Lastly, only live-born deliveries were included in this study, in recognition of the singular impact that experiencing a pregnancy loss or still-birth may have on mental health.

Limitations of this study include the use of medical encounter data, which may be subject to coding accuracy and quality. The ICD-10-CM diagnosis codes utilized to identify mental health conditions have not been validated, but we attempted to improve accuracy of capture through our requirement of 1 inpatient or 2 outpatient diagnoses on separate days. The study observation period (2016–2022) included the COVID-19 pandemic, which may have had impacts on the prevalence and detection of perinatal mental health conditions. ^22 , 23^

Additionally, the prevalence of mental health conditions only reflects those actively seeking care or otherwise engaged in the health care system. Many service members may not seek health care due to stigma, despite access to these resources. ^24 - 26^ A recent GAO report ^27^ found that only 52% of U.S. service women who delivered at a military hospital or clinic received recommended perinatal mental health screenings, increasing risk of under-diagnosis.

The true prevalence of mental health conditions among ADSW before and during the perinatal period is likely much larger than reported in this study. Future research should include prospective screening studies to identify ADSW who are not seeking care for mental health but who may meet diagnostic criteria for a mental health diagnosis. Lastly, these findings are not completely generalizable to all ADSW because those excluded were more likely of younger ages and junior enlisted members, resulting in a study population biased towards slightly older ADSW with more time in service.

This study highlights the prevalence of perinatal mental health conditions among military sub-populations. ADSW have unique mental health *and* reproductive health needs as a result of stressors inherent to military life, including, but not limited to, potential (and sometimes sudden) deployment and engagement in armed conflict. ^28 , 29^

Mental health directly affects physical health. Military service members who are mentally fit are more likely to perform their duties efficiently, enhancing operational readiness. Mental health issues can lead to early returns from deployment, which can affect unit mission and cohesion. Ten percent of all aeromedical evacuations during operations Enduring Freedom, Iraqi Freedom, and New Dawn were due to psychiatric reasons. ^30^ Service member retention is also linked to mental health, with those experiencing mental health conditions less likely to remain in the military than those not experiencing mental health conditions. ^31^

Enhanced screening and targeted management of mental health conditions may be a mechanism to improve service member retention and decrease adverse pregnancy outcomes among this population. Future research should employ qualitative methods to further explain differences in prevalence by demographic factors. Prospective studies are needed to evaluate effective screening practices and interventions for perinatal mental health conditions among ADSW.
